# A Taxonomy of Behavior Change Techniques for Improving Medication Adherence in Primary Open-Angle Glaucoma

**DOI:** 10.1155/joph/9917724

**Published:** 2025-03-26

**Authors:** Shervonne Poleon, Michael Twa, Yu-Mei Schoenberger-Godwin, Matthew Fifolt, Lyne Racette

**Affiliations:** ^1^Department of Ophthalmology and Visual Sciences, Heersink School of Medicine, University of Alabama at Birmingham, Birmingham, Alabama, USA; ^2^Department of Optometry and Vision Science, School of Optometry, University of Alabama at Birmingham, Birmingham, Alabama, USA; ^3^College of Optometry, University of Houston, Houston, Texas, USA; ^4^Division of Preventive Medicine, Heersink School of Medicine, University of Alabama at Birmingham, Birmingham, Alabama, USA; ^5^Department of Health Policy and Organization, School of Public Health, University of Alabama at Birmingham, Birmingham, Alabama, USA

**Keywords:** BCT, glaucoma, intervention, medication adherence, POAG, taxonomy

## Abstract

Many interventions aiming to improve medication adherence in primary open-angle glaucoma (POAG) have yielded equivocal findings. This equivocacy has been attributed to several factors, including limited incorporation of health behavior theory and patient preference into intervention design. In this study, we performed a literature review of interventions aiming to improve medication adherence in POAG to develop a taxonomy of behavior change techniques (BCTs). Eligible studies measured medication adherence using electronic monitors for a minimum of 3 months. For each study, we evaluated the effectiveness of the BCTs, their basis in health behavior theory, and their usefulness in day-to-day management of POAG by surveying a sample of patients and providers. Twelve studies were included. BCTs included knowledge shaping (education), prompts (reminders), behavioral rehearsal (eye drop instillation training), and pharmacological support (combination monotherapy vs. polytherapy). Knowledge shaping, prompts, health coaching, and motivational interviewing led to an improvement in medication adherence and were perceived as being most useful in day-to-day management of POAG. Taxonomies of BCTs can help researchers to improve the design and effectiveness of interventions for improving medication adherence in POAG.

## 1. Introduction

Primary open-angle glaucoma (POAG) is an optic neuropathy characterized by connective tissue remodeling, retinal damage, and visual field defects [1]. Over 75 million people worldwide live with POAG [2, 3], which is primarily managed through daily instillation of hypotensive eye drops [4]. Adherence to prescribed hypotensive therapy delays POAG progression [5], yet medication adherence is often poor [6]. In POAG, several interventions for improving medication adherence have demonstrated success [7–11]. However, there is a dearth of compelling evidence for the recommendation of specific strategies [12].

The lack of empirical evidence needed to determine which interventions may be most beneficial to which patients can be attributed to several factors, including interventions' limited reliance on health behavior theory [13] and their insufficient incorporation of patients' values and treatment preferences. Behavior change techniques (BCTs), which are specific, reproducible, and reliable strategies for eliciting behavior change [14], can help researchers to be more intentional about intervention design, which may ultimately improve intervention effectiveness [15]. BCTs are the active components within interventions that specifically aim to change behavior. By comparison, interventions are broader programs or strategies designed to bring about behavior change and often incorporate multiple BCTs. In this study [16], we aimed to develop a taxonomy of BCTs for improving medication adherence in POAG.

## 2. Methods

### 2.1. Eligibility Criteria

In this review, eligible studies were required to deliver an intervention for improving medication adherence during the implementation phase. This phase describes the period between the moment patients fill their first prescription (initiation) and the moment they stop using their medication [17]. To gather the most objective data, eligible studies were also required to measure medication adherence using electronic monitors as opposed to self-report or pharmacy claims. Lastly, to minimize the Hawthorne effect, which describes a change in research participants' behavior when under observation [18], we limited our review to studies that measured medication adherence for 3 months or more.

### 2.2. Search Strategy

Eligible studies were identified by searching Embase, JSTOR, PubMed, Directory of Open Access Journals, Scopus, Science Direct, CINAHL, PsychInfo, SAGE journals, Cochrane Central Register of Controlled Trials, Meta Register of Controlled Trials, Clinical Trials.gov, and WHO International Clinical Trials Registry. Searches were performed using the following key words: glaucoma, compliance, adherence, persistence, technique, strategy, intervention, program, electronic, MEMS, monitor, objective, dosing, medication, medication-use, increase, and improve. Truncation and Boolean operators were used. No date limiters were applied, and the gray literature (nonpeer-reviewed material such as government report or dissertation) was excluded. Searches were performed between November 2020 and March 2021.

### 2.3. Screening

Eligible studies were imported into Covidence (Veritas Health Innovation, Melbourne, Australia) for screening, risk of bias assessment, and data extraction. Two reviewers (SP and LR) independently screened all studies. Reviewers were not masked to the names of the authors or their affiliations. All disagreements between reviewers were resolved through discussion.

### 2.4. Risk of Bias Assessment

Two reviewers (S.P. and L.R.) independently assessed each study's risk of bias during sequence generation, allocation concealment, blinding, and outcome reporting. The Cochrane risk of bias assessment tool [19] was used to classify the risk of bias as low, unclear, or high. All disagreements were resolved through discussion.

### 2.5. Data Extraction-Effectiveness and Basis in Health Behavior Theory

For each study, the investigators identified the intervention delivered in the study and isolated the BCTs used in each intervention using an extensive hierarchically structured taxonomy of 93 distinct BCTs used across health conditions [14, 15]. The effectiveness of each BCT was operationalized as whether or not it led to an improvement in the medication adherence rate, therapeutic coverage (percentage of time with adequate medication use), number of administered doses, or number of adherent days. For each BCT, the reviewers searched the manuscript text to determine whether the authors provided theoretical justifications for its use in the intervention.

### 2.6. Perceived Utility

To assess the utility of each BCT in day-to-day management of POAG, patients and providers were recruited through the University of Alabama at Birmingham (UAB) Heersink School of Medicine to participate in a survey. Eligible patients were required to be above age 18 and have visual acuity better than 20/40 at baseline. Eligible providers needed to have an active 2 year history of treating POAG. Survey respondents used a semistructured questionnaire with a four-point Likert-type scale (Not Useful at All, Not Useful, Useful, and Very Useful) to quantify the usefulness of each BCT and provide qualitative judgements for their opinion (Appendix 1 and Appendix 2). The glaucoma treatment compliance assessment tool (GTCAT) [20] was also used to assess patients' perspectives on POAG [21]. All questionnaires were administered from September 2021 to October 2021. Study approval was obtained from the UAB Institutional Review Board. All aspects of the study adhered to HIPAA regulations and the tenets of the Declaration of Helsinki.

## 3. Results

**Figure 1:**
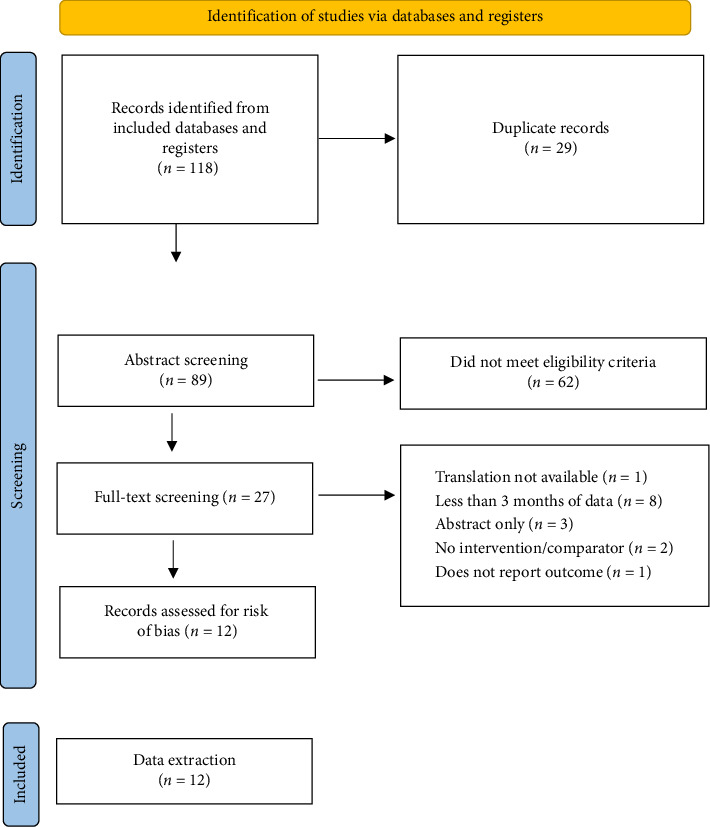
PRISMA flow diagram. The PRISMA diagram showing the number of screened, excluded, and eligible studies. PRISMA preferred reporting items for systematic reviews and meta-analyses.

The search yielded 118 studies, of which 12 were eligible (Figure 1). Studies were excluded due to failure to meet eligibility criteria (*n* = 72), being duplicates (*n* = 29), not having full text available (*n* = 3), not having an English language translation available (*n* = 1), and not reporting the outcome of interest (*n* = 1). Two studies had low risk for all potential sources of bias [22, 23], and five had either low or unclear risk for all potential sources of bias [8, 11, 24–26] (Table 1). Five studies had high risk of bias from at least one source [9, 27–30].

Interventions delivered the following BCTs: knowledge shaping (education) [8, 11, 23, 25, 27, 28], behavioral rehearsal (eye drop instillation training) [8, 11], prompts (alarms and reminders) [9, 11, 24, 25, 29–31], and pharmacological support (combination monotherapy vs. polytherapy) [26]. Several interventions were complex and delivered a combination of BCTs (Table 2) [11, 25, 27, 30]. One such intervention was health coaching, which is a patient-centered approach for eliciting behavior change based on patient values and goals. Health coaching was delivered in three studies [22, 25, 30]. In one study, health coaching included personal health plans, sessions to explore personal health goals, open-ended questions, and reflective listening techniques to address participants' health values [22].

Motivational interviewing (MI), a counseling technique aimed at reducing ambivalence [32], is also not classified as a BCT and is more akin to a complex intervention as it incorporates several different strategies. MI was delivered in four studies [27–29]. In one study, MI consisted of one-on-one meetings and phone calls with a trained glaucoma educator. Sessions reviewed medication adherence, barriers, side effects, and treatment questions. Glaucoma educators were trained to identify and address habits, beliefs, and emotions affecting adherence [28]. In a second study, MI comprised assessments of patients' thoughts and values, personalized discussions to address key glaucoma-related topics based on the patient knowledge level, and techniques to improve self-efficacy and medication use [27]. In a third study, participants receiving MI received in-person meetings and follow-up phone calls with an occupational therapist and reviewed eye drop use, barriers to adherence, side effects, treatment questions, and readiness for behavior change. During each session, MI strategies were applied to support adherence and behavior change [29]. The final study delivered MI techniques within a larger health coaching intervention but did not expound on them [22].

### 3.1. Effectiveness

Six studies reported an increase in medication adherence [9, 25, 26, 28–30] (Table 2). In one study, knowledge shaping and MI led to an increase in the percentage of days on which medication was taken as prescribed (*p*=0.032) [28]. In two others [9, 29], prompts were associated with higher medication adherence (*p* < 0.05). A fourth study providing pharmacological support reported an increase in the percentage of days on which patients adhered to dosing regimens (*p* < 0.001) [26]. In the two final studies, health coaching and prompts [25], as well as a combination of prompts, behavioral rehearsal, health coaching, and MI [30], led to an increase in the proportion of doses taken on schedule (*p* < 0.001). Overall, pharmacological support was effective in the one intervention in which it was delivered (1/1) [26], prompts were effective in 4/6 [9, 25, 29, 30], health coaching was effective in 2/3 [25, 30], knowledge shaping was effective in 2/5 [25, 30], and MI was effective in 1/3 interventions in which it was delivered [28].

Several studies did not report an improvement in medication adherence. Among these, the lack of a significant improvement was attributed to factors such as high rates of medication adherence prior to the intervention [22, 27], high rates of study attrition among patients with poorer adherence who may have eventually demonstrated an improvement after the intervention [8], short study duration [8], poor fidelity of the intervention as delivered in comparison to the planned delivery [27], possibly inaudible alarms [24], and the potential reduction in patients' focus on adherence due to increased monitoring by clinicians during the study [11].

### 3.2. Basis in Health Theory

Aside from one health coaching intervention that used the Health Decision Model during its design [30], no studies explicitly relied on health behavior theory during intervention design. While not a health behavior theory, motivational interventional theory was used to design two interventions that delivered this BCT [27, 28]. These studies cited its successful use by primary care practitioners to promote healthy behaviors across chronic diseases as a rationale for using this approach. During intervention design, these two studies also relied on the theoretical domain framework, a comprehensive framework that integrates multiple psychological theories to understand and identify the important domains involved in behavior change [27].

### 3.3. Perceived Utility

Table 3 shows the characteristics of patients and providers who evaluated the BCTs. For patients (*n* = 13), mean age was 70.9 ± 9.1 years. Median medication adherence was 74.2% (IQR = 38.9). Black patients comprised 69.2% of the sample, and male patients comprised 54%. Providers (*n* = 5) were predominantly male (80%) and White (60%) and had a mean of 12.6 years of experience. Among patients, knowledge shaping (median, IQR = 4.0, 0.25), health coaching (3.0, 2.0), and prompts (3.0, 2.0) were perceived as most useful in day-to-day management of POAG, compared to MI (4.0, 0.0), prompts (3.7, 0.33), and knowledge shaping (3.5, 0.25) among providers (Table 4). Patients with higher POAG knowledge preferred knowledge shaping (*p*=0.03), while patients with higher perceived POAG severity preferred prompts (*p*=0.02), behavioral rehearsal (*p*=0.003), and MI (*p*=0.003) (Table 5). Patients with a lower education level preferred behavioral rehearsal (*p*=0.03) and MI (*p*=0.03). Black patients preferred pharmacological support (*p*=0.008) and MI (*p*=0.03).

## 4. Discussion

The lack of empirical evidence needed to determine which interventions may be most beneficial to which patients is a critical gap in the POAG interventional landscape. We aimed to address this gap by developing a taxonomy of evidence-based BCTs for improving medication adherence. BCTs included prompts, knowledge shaping, behavioral rehearsal, and pharmacological support. While not BCTs, health coaching and MI were delivered in several studies. Prompts, knowledge shaping, health coaching, and MI were most effective in improving medication adherence and were considered most useful in improving day-to-day POAG management.

Our finding that prompts were among the most effective and highly-ranked BCTs was not surprising, as reminders and alarms have been lauded for their utility, particularly among patients with busy schedules [33]. Research also indicates that there may be a learning effect, as their benefit can persist for months after discontinuation [34]. This, along with their low-cost and simplicity, may explain their frequent use in behavioral interventions. Knowledge shaping was also effective and highly-ranked and has been shown to also improve both medication adherence and medication persistence [35, 36]. In particular, knowledge about potential future vision loss [37] and belief in treatment efficacy [38] have been identified as principal drivers of behavior change. Knowledge-shaping BCTs that include these components may be especially effective.

While few studies in this review explicitly stated a reliance on health behavior theory during their design, all BCTs that led to an increase in medication adherence had some theoretical basis for their effectiveness. For instance, by simplifying the dosing regimen, it is possible that pharmacological support led to a reduction in perceived treatment barriers-a construct of the health belief model (HBM) [21]. This model asserts that behavior is shaped by factors such as individual factors, personal beliefs, and disease-specific knowledge. In this vein, it is likely that knowledge shaping may have effected behavior change through the knowledge construct of the HBM. It is also arguable that both eye drop instillation training and prompts may have provided behavioral reinforcement, which is a construct described by the social cognitive theory (SCT). This theory argues that learning is related to observing others' behavior and understanding the consequences of this behavior [39]. Several studies that did not meet the inclusion criteria of this review have incorporated health theories such as SCT [40, 41] and HBM [42, 43]. Other health behavioral models that have been incorporated into study designs include the theory of planned behavior [44], self-determination theory [45], and the transtheoretical model [46]. By presenting these models, we aim to highlight their usefulness in understanding health behaviors, developing precise and measurable interventions, and guiding targeted and effective behavior change.

In this study, complex health interventions, namely, MI and health coaching, were highly ranked by patients and providers alike. Both interventions have been associated with improved medication adherence [47] and patient engagement [29] and may be scalable tools for increasing acceptance for treatment. As complex interventions are comprised of multiple BCTs, a notable challenge in replicating them is the often inadequate and unsystematic description of their constituent elements [48], which complicates researchers' understanding of how to reliably implement them [15]. Ensuring good design, fidelity, and reporting of complex health interventions is essential for their effectiveness, reproducibility, and impact. It is important for interventions to be theory-driven, address the right behavior change mechanisms, and tailor strategies to patient needs and real-world contexts. Standardizing training and protocols for delivering interventions can help maintain their fidelity and replicability, ensuring that observed effects result from the intervention itself rather than variations in implementation. Lastly, comprehensive reporting of intervention methodology supports transparency, integration into systematic reviews, and scaling within healthcare systems. Taxonomies of BCTs can help to standardize this process, which could in turn, improve interventions' effectiveness.

In our assessment of patient and provider perspectives, we noted a preference for prompts among patients with higher perceived POAG severity, which could potentially be due to the elevated threat of vision loss among these patients. As this threat increases, patients may be more willing to use memory aids to maintain optimal adherence. Patients with higher perceived POAG severity, together with patients of Black race and patients with a lower education level, preferred MI. It is likely that Black patients, who are at higher risk for disease progression, as well as patients who already have more severe disease, may recognize the need for more decisive action regarding POAG management. These findings provide support for the incorporation of patient preferences and multiple BCTs into intervention design to better meet the needs of increasingly diverse clinical populations.

This study has several strengths, including the development of the first BCT taxonomy specific to POAG. Other strengths include the reduction in reactivity bias by only including studies with three or more months of follow-up and the use of electronic monitoring, the most objective assessment method available. This decision has two implications that should be considered when interpreting our findings: (1) studies that used other assessment methods such as pharmacy claims were excluded [49], and (2) our sample size was small (only 12 studies met the inclusion criteria), which likely limited the number of BCTs we could identify across studies. Another limitation is that no secondary search was performed to identify studies that were published after data extraction. Lastly, when studies delivered complex interventions, individual BCTs could not be isolated and evaluated outside of the context in which they were delivered.

In this study, we developed a taxonomy of evidence based BCTs for improving medication adherence in POAG and assessed their utility among POAG patients and providers. BCTs included prompts, knowledge shaping, behavioral rehearsal, and pharmacological support. Health coaching and MI were also included as complex interventions. BCT taxonomies can help researchers to improve the design, reproducibility, and effectiveness of interventions for improving medication adherence in POAG.

## Figures and Tables

**Table 1:** Results of risk of bias assessment.

Author	Sequence generation	Allocation concealment	Participant and personnel blinding	Blinding of outcome assessors	Incomplete outcome reporting	Selective outcome reporting
Barneby and Robin [26]	Low	Unclear	Unclear	Unclear	Low	Low
Beckers et al. [8]	Unclear	Unclear	Low	Low	Unclear	Low
Boland et al. [9]	Unclear	Unclear	High	Unclear	Low	Low
Cate et al. [27]	Low	Unclear	High	Low	Low	Low
Cook et al. [28]	Unclear	Unclear	High	Unclear	High	Low
Cook et al. [29]	Low	Unclear	High	Unclear	Low	Low
Hollo and Kóth [24]	Low	Low	Unclear	Unclear	Low	Low
Lim et al. [11]	Low	Unclear	Unclear	Unclear	Low	Low
Muir et al. [30]	Unclear	Unclear	High	Unclear	Low	Low
Okeke et al. [25]	Low	Low	Unclear	Unclear	Low	Low
Richardson et al. [23]	Low	Low	Low	Low	Low	Low
Vin et al. [22]	Low	Low	Low	Low	Low	Low

**Table 2:** Study designs, populations, interventions, and outcomes.

Author	Design	Population	Sample size	Intervention (BCT)	Control	Outcome	Results
Barneby and Robin [26]	12 month rand-omized, controlled trial	Patients over 18 years old with POAG or OHT	81	*Arm 1*: fixed combination travoprost 0.004%/timo-lol 0.5% (pharmaco-logical intervention)	*Arm 2*: unfixed TRAVTIM travoprost 0.004%, timolol 0.5%	Mean % of days on which patients adhered to dosing regimens	Significant difference (TTFC vs. TRAVTIM) (*p* < 0.001)
Beckers et al. [8]	6 month multicenter rando-mized, controlled, clinical trial	Patients over 18 years old with POAG or OHT using travoprost 0.004% or fixed combination timolol 0.5% travoprost 0.004%	805; 588 analyzed	*Arm 1*: travalert dosing aid (prompts) *Arm 2*: travalert dosing aid and eye drop guide (prompts and behavioral rehearsal) *Arm 3*: travalert dosing aid, education (prompts and knowledge shaping) *Arm 4*: travalert dosing aid, drop guider, and education (prompts, behavioral rehearsal, and knowledge shaping)	NA	Change in the adherence rate	No significant difference (*p*=0.147)
Boland et al. [9]	6 month prospective study followed by randomized intervention	POAG patients over 18 years old using once daily prosta-glandin analogs	491; 70 rando-mized; 70 analyzed	Reminders (prompts)	Usual care	Change in percent adherence	Significant increase in adherence in the intervention group (*p* < 0.05)
Cate et al. [27]	8 month randomized, controlled trial	Patients with POAG and OHT or patients were glaucoma suspects	208; 114 analyzed	Education (knowledge shaping) and motivational interviewing	Usual care	Change in mean adherence	No significant difference (*p*=0.47).
Cook et al. [28]	14 week randomized, parallel group design	Patients with primary or secondary open-angle POAG who were prescribed topical mono-therapy	12; 4 analyzed	Motivational interviewing	Usual care	Change in % of days on which medi-cation was taken as prescribed	Significant increase (*T* = 2.25, *p*=0.032, *β* = 2.68)
Cook et al. [29]	12 week randomized, controlled trial	POAG patients over 18 years old on mono-therapy or combina-tion drop.	201; 177 analyzed	*Arm 1:* reminder calls (prompts) *Arm 2:* motivational interviewing	Usual care	Change in the adherence rate	Significant increase in adherence compared to usual care (*p*=0.005) (reminders)
Holló and Kóthy [24]	6 month prospective, interventional study	POAG patients using once-daily 0.004% travoprost	39; 34 analyzed	Travoprost audible alarm (prompts)			No significant change (*p*=0.059)
Lim et al. [11]	5 month rando-mized, controlled trial	POAG or ocular hypotensive patients over 18 years old	80; 77 analyzed	Education (knowledge shaping), reminders (prompts: Monthly automated telephone calls), and eye drop instillation training (behavioral rehearsal)	Usual care	1. Change in the adherence rate 2. Change in thera-peutic coverage	1. No 2. No significant group difference (*p*=0.704)
Muir et al. [30]	6 month randomized, controlled trial	Patients with medically treated POAG who reported poor adherence	200; 192 analyzed	Education (knowledge shaping), health coaching, and reminders (prompts)	Education regarding general eye health (knowledge shaping)	Mean proportion prescribed doses taken on schedule	Significant change in proportion of doses taken on schedule (0.85 vs. 0.62, *p* < 0.0001).
Okeke et al. [25]	3 month randomized controlled trial	POAG, OHT, angle-closure glaucoma, or glaucoma suspect patients 18 years or older using topical prosta-glandin analogs	66	Education (knowledge shaping), health coaching, and reminders (prompts)	Usual care	Change in medi-cation adherence (pro-portion of scheduled doses taken)	Significant change in the inter-vention group (*p*=0.001)
Richardson et al. [23]	3 month prospective study	Patients over 18 years old with POAG, OHT, or NTG receiving once-daily mono-therapy	26; 19 analyzed	Adult-centered education (knowledge shaping)	NA	1. Change in proportion of days with correct number of doses taken 2.Change in percent adherence	No significant difference pre- and post-adherence
Vin et al. [22]	6 month prospective pilot study	POAG patients taking at least one topical eye medication	4	Health coaching and motivational interviewing	Usual care	Change in proportion of prescribed doses taken on schedule	No significant difference (no *p* values provided)

Abbreviation: BCT, behavior change technique.

**Table 3:** Patient and provider characteristics.

**Patients (*N* = 13)**	

Age (years), mean ± SD	70.9 ± 9.1

Female gender (%)	54

*Race*	*Percentage (%)*
Black	69.2
White	30.8

*Highest education level*	*Percentage (%)*
High school	15.4
Some college (bachelor's degree)	69.2
Graduate or professional degree	15.4

*Income level*	*Percentage (%)*
Less than $40,000	15.4
$40–$79,999	53.8
$80–$99,999	7.7
More than $100,000	7.7
Not reported	15.4

*Number of comorbidities*	*Percentage (%)*
0–1	38.4
2–4	53.8
5 or more	7.7
Medication adherence, median (IQR)	74.2 (38.9)
*N* ocular medications, mean ± SD	1.4 ± 0.7

*GTCAT subscale scores*	*Median (IQR) [min, max]*
Perceived beliefs	4 (1.0) [1, 5]
Perceived barriers	5 (0.0) [1, 5]
Cues to action	2.5 (1.1) [1, 5]
Self-efficacy	4.2 (0.5) [1, 5]
Perceived severity	3 (0.5) [1, 5]
Perceived susceptibility	4 (2.0) [1, 5]
POAG knowledge	4.4 (0.85) [1, 5]

**Providers (*N* = 5)**	

Male gender (%)	80

*Age group*	*Percentage (%)*
25–34 years	40
35–44 years	20
45, 50, and 54 years	20
55–64 years	20

*Race*	*Percentage (%)*
White	60
Asian	20
Not reported	20

**Table 4:** Taxonomy of BCTs.

BCT	Effectiveness	Theoretical basis	Usefulness (Patients)	Usefulness (Providers)	Subjective judgments
Education	Significant improvement in 2 out of 6 studies	None stated	Rank = 1 Median = 4.0 IQR = 0.25	Rank = 3 Median = 3.5 IQR = 0.25	“The program would educate me about glaucoma, how does it start, what kicks it off? can it be prevented?”
Health coaching	Significant improvement in 2 out of 3 studies	None stated	Rank = 2 Median = 3.0 IQR = 2.0	Rank = 5 Median = 2.7 IQR = 2.0	“Many patients take what a doctor says without question. If they understand something prior to the visit, they would be more apt to follow directions”
Reminders	Significant improvement in 4 out of 7 studies	None stated	Rank = 3 Median = 3.0 IQR = 2.0	Rank = 2 Median = 3.7 IQR = 0.33	“Sometimes our schedules are so full, we forget to administer the drops”
Eye drop instillation training	Significant improvement in 1 out of 3 studies	None stated	Rank = 4 Median = 3.0 IQR = 2.0	Rank = 5 Median = 2.7 IQR = 0.67	“It's useful because one would be able to know if they use too much medication or less because it was taken incorrectly”
Motivational interviewing	Significant improvement in 2 out of 4 studies	None stated	Rank = 5 Median = 3.0 IQR = 2.0	Rank = 1 Median = 4.0 IQR = 0.0	“Each person has their own method of handling medical issues. Explain the disease management and let the person learn to cope with it”
Pharmacological support	Significant improvement in 1 out of 1 study	None stated	Rank = 6 Median = 3.0 IQR = 2.0	Rank = 4 Median = 3.0 IQR = 0.0	“I would definitely be interested in a combination of eye drops. It gets very tedious applying multiple drops sometimes”

Abbreviation: BCT, behavior change technique.

**Table 5:** Correlation values between patient characteristics and perceived BCT utility.

	Education	Planning/problem solving	Reminders	Instillation training	Pharmaco-logical support	Motivational interviewing
Perceived barriers	−0.189	0.170	−0.395	−0.060	0.345	−0.155
Perceived benefits	−0.189	−0.323	−0.368	−0.288	0.161	−0.321
Cues to action	−0.263	−0.172	−0.395	−0.204	0.169	−0.172
Glaucoma knowledge	0.603⁣^∗^	0.001	−0.036	−0.036	0.160	−0.017
Self-efficacy	0.237	−0.091	−1.00	−1.00	−0.199	−0.032
Perceived severity	0.274	0.348	0.626⁣^∗^	0.754⁣^∗∗^	0.524	0.754⁣^∗∗^
Perceived susceptibility	0.241	0.151	0.258	0.062	0.036	0.208
Age	0.089	−0.068	0.157	0.125	−0.346	0.000
Sex	0.202	−0.215	0.000	−0.110	−0.086	−0.215
Race	0.000	−0.237	−0.486	−0.545	−0.695⁣^∗∗^	−0.603⁣^∗^
Education level	−0.363	−0.504	−0.530	−0.592⁣^∗^	−0.193	−0.694⁣^∗∗^
Income level	−0.049	−0.090	−0.461	−0.284	−0.406	−0.320
Adherence rate	−0.185	−0.241	−0.419	−0.404	0.177	−0.532

⁣^∗^Significant correlation at the 0.05 level (two-tailed).

⁣^∗∗^Significant correlation at the 0.01 level (two-tailed).

## Data Availability

The data that support the findings of this study are available from the corresponding author upon reasonable request.

## Supporting Information

Supporting Information 1 Appendix 1. Copy of questionnaire issues to patient participants to facilitate their evaluation of the different BCTs provided by the interventions included in this study.

Supporting Information 2 Appendix 2. Copy of questionnaire issues to provider participants to facilitate their evaluation of the different BCTs provided by the interventions included in this study.
