# Supplementary material for: A Taxonomy of Behavior Change Techniques for Improving Medication Adherence in Primary Open-Angle Glaucoma
Source: J Ophthalmol. 2025 Mar 26;2025:9917724. doi: 10.1155/joph/9917724 (PMC11964726; doi:10.1155/joph/9917724)
Supplement: Supporting Information 1 — Appendix 1. Copy of questionnaire issues to patient participants to facilitate their evaluation of the different BCTs provided by the interventions included in this study. [file 9917724.f1.docx]

**INTERVENTIONS FOR IMPROVING MEDICATION ADHERENCE IN GLAUCOMA – PATIENT QUESTIONNAIRE**

**Below are several statements about glaucoma and your experience with glaucoma medications. Please read each statement carefully and circle the response that best represents your opinion.**

1. My personal knowledge of the symptoms of glaucoma is excellent.

Disagree a lot Disagree a little No Opinion Agree a little Agree a lot.

2. A person can have glaucoma and not know it.

Disagree a lot Disagree a little No Opinion Agree a little Agree a lot.

3. Eye pain is a common symptom of glaucoma.

Disagree a lot Disagree a little No Opinion Agree a little Agree a lot.

4. Glaucoma treatments can prevent future vision loss.

Disagree a lot Disagree a little No Opinion Agree a little Agree a lot.

5. Vision lost from glaucoma is permanent.

Disagree a lot Disagree a little No Opinion Agree a little Agree a lot.

6. I completely agree with my doctor’s diagnosis of glaucoma in my eye(s).

Disagree a lot Disagree a little No Opinion Agree a little Agree a lot.

7. I have lost none of my vision due to glaucoma.

Disagree a lot Disagree a little No Opinion Agree a little Agree a lot.

8. Losing the same amount of vision in the next 5 years as I have in the past 5 would not affect my quality of life.

Disagree a lot Disagree a little No Opinion Agree a little Agree a lot.

9. Over the last month I have not missed taking my eye drops.

Disagree a lot Disagree a little No Opinion Agree a little Agree a lot.

10. Sometimes I forget to use my drops.

Disagree a lot Disagree a little No Opinion Agree a little Agree a lot.

11. Sometimes I fall asleep before dosing time.

Disagree a lot Disagree a little No Opinion Agree a little Agree a lot.

12. Sometimes the drops aren’t with me when it is time to take them.

Disagree a lot Disagree a little No Opinion Agree a little Agree a lot.

13. Sometimes I am out of drops.

Disagree a lot Disagree a little No Opinion Agree a little Agree a lot.

14. I need assistance putting drops in my eyes.

Disagree a lot Disagree a little No Opinion Agree a little Agree a lot.

15. I suffer from side effects when using my drops.

Disagree a lot Disagree a little No Opinion Agree a little Agree a lot.

16. My eye drops cause me no pain or discomfort.

Disagree a lot Disagree a little No Opinion Agree a little Agree a lot.

17. My eye drops are difficult to use.

Disagree a lot Disagree a little No Opinion Agree a little Agree a lot.

18. I think I will go blind in 10 years if I DO NOT use my eye drops.

Disagree a lot Disagree a little No Opinion Agree a little Agree a lot.

19. A friend or family member’s experience with eye drops has encouraged me to use my eye drops.

Disagree a lot Disagree a little No Opinion Agree a little Agree a lot.

20. I can place the eye drops into my eye correctly without any assistance.

Disagree a lot Disagree a little No Opinion Agree a little Agree a lot.

21. There are things I can do to control my glaucoma.

Disagree a lot Disagree a little No Opinion Agree a little Agree a lot.

22. I can afford my eye drops.

Disagree a lot Disagree a little No Opinion Agree a little Agree a lot.

23. I use reminders to take my eye drop medications.

Disagree a lot Disagree a little No Opinion Agree a little Agree a lot.

24. My doctor answers my questions.

Disagree a lot Disagree a little No Opinion Agree a little Agree a lot.

25. I am happy with my eye doctor.

Disagree a lot Disagree a little No Opinion Agree a little Agree a lot.

26. My overall health is excellent.

Disagree a lot Disagree a little No Opinion Agree a little Agree a lot.

27. Over the past 4 weeks I have never felt blue, downhearted, or depressed.

Disagree a lot Disagree a little No Opinion Agree a little Agree a lot.

**BLOCK 2: PROGRAMS FOR IMPROVING EYEDROP USE**

**Below are seven programs that can help patients to improve their eyedrop use. Think about the problems and challenges that you experience from day to day when you use your glaucoma eyedrops and circle the response that best describes how useful each program is to you. Use the lines provided to give a brief explanation.**

1. An educational program that would help you to:

- Better understand glaucoma and how it leads to blindness.
- Better understand how glaucoma should be treated and managed.
- Better understand your test results and treatment recommendations from your doctor.
- Better understand the importance of using eye drops and how they cause side effects.

1= not useful at all 2=not useful 3=useful 4=very useful

___________________________________________________________________________

___________________________________________________________________________

___________________________________________________________________________

1. A health-coaching program that:
   - Helps you to become more confident speaking with your doctor and clinical staff.
   - Helps you to become more involved in your treatment discuss and problems you experience.
   - Helps you to become more confident challenging the healthcare system (asking questions, discussing treatment alternatives and preferences).

1= not useful at all 2=not useful 3=useful 4=very useful

___________________________________________________________________________

___________________________________________________________________________

___________________________________________________________________________

1. An eyedrop instillation skill program that:
   - Helps you to feel more confident about being able to correctly instill your eyedrops.
   - Provides tips, guides, and training on how to correctly instill your eyedrops.
   - Provides devices that can help you to properly instill your eye drops.

1= not useful at all 2=not useful 3=useful 4=very useful

___________________________________________________________________________

___________________________________________________________________________

___________________________________________________________________________

1. A memory-based program that uses audio or visual reminders to use your eyedrops and helps you to fit your eyedrops into your daily schedule.

1= not useful at all 2=not useful 3=useful 4=very useful

___________________________________________________________________________

___________________________________________________________________________

___________________________________________________________________________

1. A pharmaceutical program that provides drug combinations and alternative eyedrop brands. The drug combinations (2 in 1) would reduce your overall number of eyedrop medications. The alternative brands may reduce the number of times you would need to instill your drops each day.

1= not useful at all 2=not useful 3=useful 4=very useful

___________________________________________________________________________

___________________________________________________________________________

___________________________________________________________________________

1. A health counseling (motivational interviewing) program that:
   - Helps you to set goals for managing your glaucoma and create a personalized health plan.
   - Helps you to recognize any habits, beliefs, or feelings that interfere with using your eyedrops as prescribed by your doctor.
   - Provides problem-solving skills for overcoming habits or beliefs that interfere with your eyedrop use.

1= not useful at all 2=not useful 3=useful 4=very useful

___________________________________________________________________________

___________________________________________________________________________

___________________________________________________________________________

1. A health monitoring program that provides devices and software that track your eyedrop use and shares this information with your doctor.

1= not useful at all 2=not useful 3=useful 4=very useful

___________________________________________________________________________

___________________________________________________________________________

___________________________________________________________________________

-END OF QUESTIONNAIRE-
