# Supplementary material for: A Taxonomy of Behavior Change Techniques for Improving Medication Adherence in Primary Open-Angle Glaucoma
Source: J Ophthalmol. 2025 Mar 26;2025:9917724. doi: 10.1155/joph/9917724 (PMC11964726; doi:10.1155/joph/9917724)
Supplement: Supporting Information 2 — Appendix 2. Copy of questionnaire issues to provider participants to facilitate their evaluation of the different BCTs provided by the interventions included in this study. [file 9917724.f2.docx]

**INTERVENTIONS FOR IMPROVING MEDICATION ADHERENCE IN GLAUCOMA – PROVIDER QUESTIONNAIRE**

**Think about the problems and challenges that patients experience from day to day when they use their glaucoma eyedrops. Below are seven different programs that can help patients to improve their eyedrop use. Pease rate each program from 1-4 depending on how much you believe it would help patients to improve or keep up their eyedrop use (1 = not useful at all, 2 = not useful, 3 = useful, 4 = very useful). In addition, please use the lines provided to explain why this program is useful or not useful.**

1. An educational program that would help patients to:

- Better understand glaucoma and how it leads to blindness.
- Better understand how glaucoma should be treated and managed.
- Better understand their test results and treatment recommendations from their doctor.
- Better understand the importance of using eye drops and how they cause side effects.

1= not useful at all 2=not useful 3=useful 4=very useful

___________________________________________________________________________

___________________________________________________________________________

___________________________________________________________________________

1. A health-coaching program that:
   - Helps patients to become more confident speaking with their doctor and clinical staff.
   - Helps patients to become more involved in their treatment discuss and problems patients experience.
   - Helps patients to become more confident challenging the healthcare system (asking questions, discussing treatment alternatives and preferences).

1= not useful at all 2=not useful 3=useful 4=very useful

___________________________________________________________________________

___________________________________________________________________________

___________________________________________________________________________

1. An eyedrop instillation skill program that:
   - Helps patients to feel more confident about being able to correctly instill their eyedrops.
   - Provides tips, guides, and training on how to correctly instill their eyedrops.
   - Provides devices that can help patients to properly instill their eye drops.

1= not useful at all 2=not useful 3=useful 4=very useful

___________________________________________________________________________

___________________________________________________________________________

___________________________________________________________________________

1. A memory-based program that uses audio or visual reminders to use their eyedrops and helps patients to fit their eyedrops into their daily schedule.

1= not useful at all 2=not useful 3=useful 4=very useful

___________________________________________________________________________

___________________________________________________________________________

___________________________________________________________________________

1. A pharmaceutical program that provides drug combinations and alternative eyedrop brands. The drug combinations (2 in 1) would reduce their overall number of eyedrop medications. The alternative brands may reduce the number of times patients would need to instill their drops each day.

1= not useful at all 2=not useful 3=useful 4=very useful

___________________________________________________________________________

___________________________________________________________________________

___________________________________________________________________________

1. A health counseling (motivational interviewing) program that:
   - Helps patients to set goals for managing their glaucoma and create a personalized health plan.
   - Helps patients to recognize any habits, beliefs, or feelings that interfere with using their eyedrops as prescribed by their doctor.
   - Provides problem-solving skills for overcoming habits or beliefs that interfere with their eyedrop use.

1= not useful at all 2=not useful 3=useful 4=very useful

___________________________________________________________________________

___________________________________________________________________________

___________________________________________________________________________

1. A health monitoring program that provides devices and software that track patients’ eyedrop use and shares this information with their doctor.

1= not useful at all 2=not useful 3=useful 4=very useful

___________________________________________________________________________

___________________________________________________________________________

___________________________________________________________________________

-END OF QUESTIONNAIRE-
